# Toll like receptor signaling in “inflammaging”: microRNA as new players

**DOI:** 10.1186/1742-4933-10-11

**Published:** 2013-03-19

**Authors:** Fabiola Olivieri, Maria Rita Rippo, Francesco Prattichizzo, Lucia Babini, Laura Graciotti, Rina Recchioni, Antonio Domenico Procopio

**Affiliations:** 1 Department of Clinical and Molecular Sciences, Università Politecnica delle Marche, Ancona Via Tronto 10/A, Ancona, 60020, Italy; 2 Center of Clinical Pathology and Innovative Therapy, I.N.R.C.A. National Institute, Ancona, Italy

**Keywords:** MicroRNA, TLR, Aging, Cellular senescence, Inflammaging, SASP, Endothelial cells, Innate immunity cells

## Abstract

The age-related changes of immune system functions are complex phenomena incompletely understood. The acquired immune system shows a functional decline in ability to respond to new pathogens during aging, whereas serum levels of inflammatory cytokines are increased with age. The source of this age-related systemic chronic inflammation, named inflammaging, was mainly attributed to the progressive activation of immune cells over time. However, recent studies have shown that the process of cellular senescence can be an important additional contributor to chronic inflammation, since senescent cells acquire a phenotype named “senescence-associated secretory phenotype” (SASP), characterized by the enhanced secretion of many inflammation modulators. Pathogen-associated molecular pattern receptors, in particular Toll-like receptors (TLRs), are key molecules in the response of innate immunity cells to pathological stimuli. An intriguing and innovative hypothesis is that the dysfunction of TLRs signaling and the acquisition of SASP can be two interconnected phenomena. The TLR family, including receptors and co-effector molecules, do not show a consistent age-dependent change across model systems. However, there is evidence for impaired downstream signaling events, including inhibition of positive and activation of negative modulators of TLR signaling. MicroRNAs (miRNAs) are a newly discovered class of gene regulators acting as post-transcriptional repressors of a number of genes. The miRNA property to finely-tune gene expression makes them right for immune system regulation, which requires precise control for proper activity. We reviewed evidences suggesting that miRNAs can modulate TLR signaling mainly by three different mechanisms: 1) miRNAs can directly target components of the TLR signaling system, 2) miRNA expression can be directly regulated by TLRs pathway activation and 3) miRNAs can directly activate the RNA-sensing TLRs, like TLR-8, in humans. We also reviewed how TLR signaling is modulated by miRNAs during aging, and how an impaired miRNAs/TLR signaling interaction in immune system cells and related cells, i.e. endothelial cells and adipocytes, can contribute to inflammaging observed in normal aging. Interestingly, this impairment appears accelerated in presence of the majors age-related diseases, such as cardiovascular diseases, diabetes, neurodegenerative diseases and cancers.

## Toll like receptors and their pathogen-derived and endogenous ligands

Activation of the innate immune system is based on the recognition of conserved microbial and viral structures by a limited number of membrane and cytosolic resident receptor molecules. Among these molecules, Toll like receptors (TLRs) are transmembrane proteins of mammalian cells implicated mainly in the recognition of bacterial and viral components [1,2]. To date, at least 11 TLRs have been described in humans. Specifically, TLR-2 can hetero-dimerize with TLR-1 or TLR-6, and either TLR-1/2 or TLR-2/6 can recognize bacterial lipoproteins, such as peptidoglycan or lipopeptide; TLR-3 recognizes viral double-stranded RNA; TLR-4 binds the endotoxin lipopolysaccharide (LPS) from Gram-negative bacteria; TLR-5 recognizes bacterial flagella proteins; TLRs-7 and −9 detect pathogenic nucleic acids; TLR-10 can hetero-dimerize with TLR-1 or TLR-2 but its ligand is currently unknown; finally TLR-11 can recognize molecules from the uro-phatogenic bacteria [2,3]. Thus, TLR −2, -4 and −6 function as the principal innate sensors for cell-wall components of gram-negative bacteria in mammals [4]. Viral nucleic acid molecules are recognized by the endosomal TLRs −3, -7, -8, and −9, by the cytosolic retinoic acid-inducible gene-like receptors (RLRs) Rig-I and Mda5, and by the dsRNA-dependent protein kinase R (Pkr) [5]. TLR-3 and the RLRs recognize double stranded RNA (dsRNA) molecules, whereas TLR −7, -8 and −9 recognize single stranded RNA (ssRNA) and DNA. The endosomal TLR-7, -8 and −9 signal act via MyD88, whereas TLR-3 uses the TIR domain containing adaptor inducing interferon-β (TRIF) molecule. Thus, all TLRs, with the exception of TLR-3, require MyD88 to induce phosphorylation of IL-1 receptor associated kinase (IRAK)-1 by IRAK4. Phosphorylated IRAK1 recruits and activates TNFR-associated factor 6 (TRAF6). TRAF6 can then activate protein kinase C (PKC), Extracellular Signal Regulated Kinase (ERK)-1/2, and Transforming Growth Factor (TGF)-β-activated kinase 1 (TAK1). TAK1 is a Mitogen Activated Protein Kinase (MAPK) able to phosphorylate p38 MAPK, c-Jun N-terminal kinase (JNK), and I-kappa kinase (IκK). Activation of IκK leads to the nuclear translocation of Nuclear Factor kappa-B (NF-κB) and subsequent transcription of genes associated with TLR activation. NF-kB is a transcriptional regulator consisting of reticuloendotheliosis (Rel) protein dimers that bind a DNA sequence motif known as kB site, and it is retained in the cytoplasm by specific kB inhibitors, such as IkBα, IkBβ, IkBγ, IkBζ, IkBNS and Bcl-3 [6,7]. Overall, pathogen-derived ligand-induced TLR engagement and signal transduction leads to activation of NF-κB and thereby to the induction of pro-inflammatory and antiviral response genes [8].

More controversial is the potential role of TLRs in the recognition of endogenous ligands and which effect this recognition might have on the consequent development of chronic inflammatory disorders. An increasing number of studies implicate TLRs as being involved in the immune response to self-molecules that may be altered or could be accumulated in non-physiologic sites or amounts. Prolonged injury induces tissue damage and generation of endogenous TLR-4 ligands able to activate TLR-4 signaling, sustaining in turn fibroblast activation [9]. For example, Herpes simplex virus type 1 (HSV-1) induces and activates TLR-2 and TLR-4 receptors at the central nervous system (CNS) both directly, through interaction of astrocytes with the pathogen, and also indirectly, by endogenous ligands produced locally, such as serum amyloid protein [10]. Further, in rheumatoid arthritis (RA), Snapin (SNAP associated protein) was identified as a novel endogenous TLR-2 ligand [11]. The extracellular matrix glycoprotein tenascin-C is an endogenous activator of innate immunity that promotes the synthesis of inflammatory cytokines via activation of TLR-4 [12]. Furthermore, the soluble form of the extracellular matrix proteoglycan decorin was demonstrated to act as an endogenous ligand of TLR −2 and −4, stimulating the production of proinflammatory molecules in human macrophages [13].

Recent findings have shown increased TLR-2 and −4 expression, signaling, ligands, and functional activation both in type 1 diabetes mellitus (T1DM) subjects and in monocytes of type 2 diabetic (T2DM) patients [14]. This increased activity of TLRs in diabetes seems to be the result of both endogenous and exogenous ligands [15]. TLR-2 knockout mouse model has been used to show the important role played by TLRs to promote the pro-inflammatory state of T1DM and the progression of diabetic nephropathy [16].

When it was tested the hypothesis that TLR-4-induced transcription patterns elicited in humans exposed to in vivo endotoxin would parallel gene expression patterns observed in trauma patients with initial non-infectious injury, a group of more than 400 genes that exhibit similar expression trends in leukocytes derived from either endotoxin-challenged subjects or trauma patients was found [17].

Overall, besides the general concept that TLRs are implicated in the recognition of bacterial and viral components, many data suggested that TLRs can also play a role in the recognition of several, not all identified, endogenous ligands. Recently, it was proposed to name all the endogenous TLRs ligands as damage associated molecular pattern molecules (DAMPs) [18]. It can be hypothesized that alterations of TRLs/ligands, both exogenous and endogenous, may contribute to the pathogenesis of human diseases, especially the major age-related diseases, such as cardiovascular diseases, diabetes, neurodegenerative diseases and cancers.

### Age-related changes in TLR expression in immune system cells

Age-related human diseases are mainly, or at least in part, due to numerous changes in the immune system which result in a significant decline in protective immunity [19]. Although it is well documented that both B and T lymphocyte compartments of the adaptive immune system deteriorate with advancing age, the impact of aging on many aspects of the innate immune response remains to be elucidated. In fact, even in the absence of an immune challenge, healthy-aged individuals have a significantly higher basal inflammatory state, characterized by increased circulating cytokines levels, including IL-6, IL-1β and TNF-α [20]. This progressive pro-inflammatory status, termed inflammaging, renders the older subjects more susceptible to a poor prognosis following systemic insults [20,21]. Inflammaging is one of the major driving forces of frailty and of common severe age-related diseases development and progression, including cardiovascular diseases, type 2 diabetes mellitus and neurodegenerative diseases [22,23]. It can be hypothesized that alteration of TLRs and/or TLR-ligands expression levels, both exogenous and endogenous, during cellular aging could contribute to such inflammation imbalance. The age-related expression of the TLR signaling pathway components have been extensively studied in cells of the innate immunity, mainly neutrophils and macrophages. The effect of aging on these cells appears to be multifaceted, affecting almost every aspect of their normal cellular function. Myeloid precursors differentiate into macrophages in the presence of growth factors such as macrophage-colony stimulating factor (M-CSF), granulocytes macrophage-colony stimulating factor (GM-CSF) and IL-3. There is a degree of ambiguity on how age can effect the generation of macrophages from their precursor. The number of monocytes in the aged and young subjects appears to be comparable; however, there is a significant decrease in macrophage precursors as well as macrophages in the bone marrow of the elderly [24].

TLRs expression in cells of innate immunity, such as macrophages, granulocytes and dendritic cells, was analyzed in vitro and in vivo, both in animal models and in humans, during aging/senescence processes, showing complex results.

### Age-related TLR expression changes in immune system cells of animal model

A number of evidences in murine models support the notion that aging do not affect the expression levels of TLR. The expression levels of TLR-2, -4 and −6, in absence of stimulation, was similar in peritoneal macrophages, lymphocytes, and macrophages from older and younger mice [25,26]. However, the sensitivity to repeated LPS exposure in cells from young and middle-aged mice is different and seems to be partly associated with the different expressions levels of TLR-2 and −4 [27]. LPS is one of the most important virulence factors of gram-negative bacteria, and plays an essential role in triggering inflammation. Endotoxin tolerance induced by repeated LPS stimulations could lead to the reprogramming of the immune system with the aim to obtain a protective role against inflammatory tissue destruction [28]. The down-regulation of TNF-α and IL-1β, the main pro-inflammatory cytokines, and the preservation of IL-10, the main anti-inflammatory cytokine, seem to confirm this hypothesis. In addition, the decreased secretions of TNF-α and IL-10 by LPS-stimulated peritoneal macrophages from middle-aged mice, indicates the impaired functions of TLR −2 and- 4 in these cells, which might be associated with the age-related changes in TLR-2 and −4 signaling transduction [26].

Moreover, total levels of TLR-1 were unaffected by age whereas TLR-2 surface expression was significantly but slightly increased in alveolar macrophages from aged mice, suggesting that intracellular TLR signaling defects, rather than TLRs expression, were responsible for the age-related decline in in vivo responsiveness to *pneumococcal pneumonia*[29]. However, TLR-4 and TLR-5 expression was reported to be higher in old rhesus macaques (*Macaca mulatta*) compared to younger animals [30].

Overall, no conclusive results can be obtained on TLRs baseline expression level in animal model during aging. However, tolerance induced by LPS repeated exposure seems to be related to age. The lack of response to the stimulation of LPS and the increased proinflammatory cytokines release observed in macrophages of aged mice suggest that TLRs signaling transduction is impaired in cells from aged animals.

### Age-related TLR expression changes in human immune system cells

Recently, the surface expression of TLRs −1, -2, -4, -5, and −6 from monocytes of younger and older individuals was quantified by flow cytometry, showing complex results. It was reported a decreased expression of TLR-1 and TLR-4, unchanged levels of TLR-2 and TLR-6 and a significant increased expression of TLR-5 in monocytes from older individuals compared with that of younger ones [31]. Basal levels of TLR-2 and TLR-4 expression were previously showed to be unaltered by age on neutrophils and monocytes of aged and young donors [32,33].

The production of age-related cytokines following stimulation with a wide range of TLR ligands has also been studied in monocytes from young and older donors. While the basal levels of IL-8 and TNF-α were equivalent between the age groups, macrophage migration inhibitory factor (MIF) had higher basal levels in monocytes from older donors [31]. In addition, a higher representation at baseline of the signaling mediator NF-κB (p65) translocated to the nucleus of monocytes from older donors was showed [31]. Interestingly, it was observed a statistically significant stimulation-specific increase in the production of IL-8 by adherent monocytes from older individuals for all TLR ligands, even if the magnitude of IL-8 production was highest following treatment with the TLR-5 ligand, flagellin [31]. Overall, even if cytokine plasma levels is increased with age, the production of IL-6 and TNF-α was decreased in macrophages from aged compared to young individuals. This apparent paradox of low cellular production and high plasma cytokine levels may be resolved by considering that with age, macrophages remain activated for longer and by multiple stimuli and have an increased lifespan [34]. Thus, the ineffective clearance of pathogens by macrophages can increase the duration of their activation and contribute to perpetuation of inflammatory responses during ageing.

In conclusion, gene transcription, protein expression and cell surface expression of members of the TLR family (receptors and co-effector molecules) of innate immunity cells do not show a consistent age-dependent change across immune system models. However, there is evidence for impaired downstream signaling events, including inhibition of positive and activation of negative modulators of TLR induced signaling events.

### Age-related changes in TLR expression in non immune system cells

Interestingly, cells that are not typically involved in the innate immune response such as T cells, B cells, epithelial cells, endothelial cells, adipocytes, cardiac cells and others, are able to produce cytokines in response to TLR ligands interaction.

It was described an age-dependent upregulation of intestinal epithelial TLR-3 expression in humans, which might contribute to rotavirus reduced susceptibility in adults [35].

We recently showed that also endothelial cells are able to produce cytokines via TLR signaling. An increased release of interleukin (IL) -1β, -1α -2, -6, -8, -10, -12, tumor necrosis factor (TNF)-α, interferon (INF)-γ and mieloperoxidase (MPO) were observed in senescent vs. younger human umbilical vein endothelial cells (HUVEC) [36]. Moreover, after LPS stimulation, an increased IL-6 release was observed. Interestingly, the strength of this effect depends on the senescent status of HUVEC (unpublished data).

Cardiac myocytes express many of the TLRs and ligands able to activate TLR-2, TLR-4, and TLR-5 inducing NF-κB activation and IL-6 and intercellular adhesion molecule (ICAM)-1 expression in vitro [37].

Interestingly, TLRs expression was also studied in hematopoietic stem cell (HSC). Selective loss of lymphopoietic potential in HSC has been seen in studies of normal aging, and could conceivably contribute to immunosenescence [38]. However, the mechanism(s) responsible for HSC aging have only begun to be elucidated [39]. Recent studies have reported epigenetic changes, accumulation of DNA damage, and increased levels of intracellular reactive oxygen species in aged HSCs. Interestingly, HSC from mice are injured by persistent exposure to small amounts of a single TLR ligand, such as LPS. Although phenotypically defined HSCs increased in LPS treated mice, their repopulating and lymphopoietic potentials dramatically declined [38]. Thus, low-grade host/pathogen interactions may be detrimental for stem cells functions over time.

Although the TLR expression modulation in adipocytes during aging has not yet been investigated, their contribution on chronic inflammation, a phenomenon named as metaflammation, was also highlighted. Recent studies suggest that adipocytes may play an important role in the physiological regulation of immune/inflammatory responses in fat deposits via TLR signaling cascades [40,41]. Adipocytes are no longer considered passive cells storing fat, but they share with macrophages many biological properties including synthesis and release of molecules regulating inflammation. Fatty acid levels, which are elevated in obesity, can induce both local and systemic inflammation via activation of TLR [42]. Thus, a positive feedback loop between local inflammation in adipose tissue and altered immune response in obesity, may contribute to the development of obesity-related metabolic complications and the inhibition of NF-kB signalling pathways could be an excellent strategy for treatment of metabolic complications in obese individuals.

### TLR signaling modulation via microRNAs

TLR signaling must be stringently regulated in order to ensure sufficient clearance of pathogens and a timely return to homeostasis after infection. MiRNAs are a newly discovered class of gene regulators which bind to the 3′ untranslated region of target mRNA and direct their post-transcriptional repression. The discovery of miRNAs has revolutionized the way we examine the regulation of gene transcription and translation. The special properties of miRNA to finely-tune gene expression naturally lends itself to immune system regulation, which requires regulatory mechanisms for proper activity.

Evidences were provided suggesting that miRNAs can modulate TLR signaling mainly by three different mechanisms: 1) miRNAs can directly target components of the TLR signaling system, 2) miRNAs expression can be directly regulated by TLRs and 3) miRNAs can directly activate the RNA-sensing Toll-like receptor.

**Figure 1:**
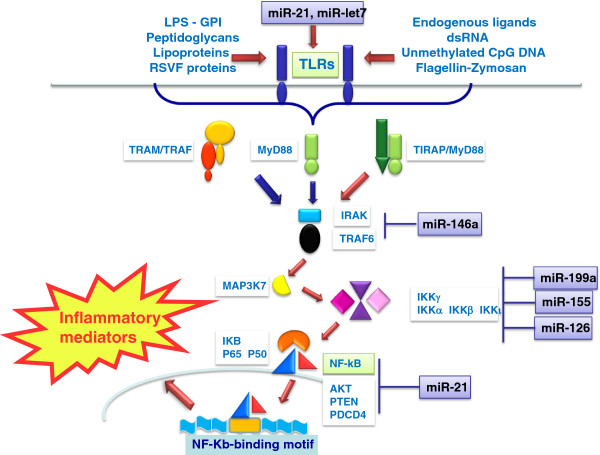
**MicroRNA (miRs) modulating TLRs pathways.** GPI = glycophosphoinositol; RSV= respiratory syncytial virus.

Regarding the first two mechanisms, it is important to note that several miRNAs have been shown to be up-regulated in response to TLR ligands, and many of them directly target components of the TLR signaling system, revealing a feedback loop control of TLR signaling activation. Specific miRNAs were reported to modulate trascription of receptors, adaptor molecules, and regulator molecules within TLR and IL-1R signaling, through positive and negative feedback loops. A trio of miRNA, such as miR-155, miR-21 and miR-146a, have proven to be key TLR signaling modulators and, importantly, these miRNas are codified by endotoxin-responsive genes [43]. The role of different miRNAs mainly involved in modulating TLRs pathways is described here and depicted in Figure 1.

### MiR-146a

MiR-146a is known to target key elements of MyD88 signaling pathway, including IRAK1 and TRAF6, modulating in turn the inflammatory responses mediated by TLR-4, TLR-2 and TLR-5 ligands [44]. Thus, a role for miR-146a in control of TLR receptor and cytokine signaling through a negative feedback regulation loop involving down-regulation of IRAK1 and TRAF6 protein levels was proposed [45]. A lot of evidence demonstrated that miR-146a is critical for endotoxin tolerance observed in in vitro cultured monocytes [46]. Interestingly, miR-146a, which negatively regulates the expression of IL-1β and IL-6, was highly expressed in aged mice as a consequence of an aberrant NF-κB binding to the miR-146a promoter [47]. As a result, the negative feedback regulation loop inducing a down-regulation of inflammation factors resulted interrupted in aged mice [47]. In macrophages isolated from aged mice the DNA methyl-transferase inhibitor and the histone deacetylase inhibitor are both able to significantly up-regulate miR-146a transcriptional activation by altering the DNA-binding activity of NF-κB. These data suggest that DNA methylation and histone acetylation are involved in the suppression of age-dependent miR-146a expression. Overall, these data indicate that the deregulated expression of miR-146a contributes to the age-associated dysfunction of macrophages in aged animals [47].

We recently reported that in endothelial cells, such as HUVEC and in circulating angiogenic cells (CACs) derived from monocytic lineage, IRAK1 protein, but not TRAF6, modulation depends on miR-146a [36]. We also observed that LPS stimulation induces an increased expression of miR-146a and that younger cells respond faster than senescent ones to LPS stimulation, suggesting a more efficient stimulus-induced response by young cells (unpublished data).

### MiR-155

MiR-155 may control the expression of IKKβ and IKKε, which leads to repression of NF-κB activation. Its expression in mature human dendritic cells (DCs), is part of a negative feedback loop, which down-modulates inflammatory cytokine production in response to microbial stimuli [48].

### MiR-21

MiR-21 targets PTEN, an inhibitor of AKT, which in turn activates NF-κB [45]. Another well established target of miR-21 is the tumor suppressor PDCD4, a proinflammatory protein that promotes activation of the transcription factor NF-κB and suppresses interleukin 10 (IL-10) [49]. Transfection of cells with a miR-21 precursor blocked NF-κB activity and promoted the production of the anti-inflammatory IL-10 in response to LPS. In addition to PTEN and PDCD4, a number of other targets of miR-21 including tropomyosin (TPM1), sprouty1 and 2, TGF-β receptor (TGFBR2) Cdc25a have been validated [50,51].

Importantly, mir-21 was reported to modulate TLR-2 signaling in mice, suggesting an in vivo role of miR-21 in suppressing TLR-2 signaling [52]. As reported for miR-146a, also miR-21 precursor transcript is up-regulated by NF-kB [53].

Furthermore, our recent findings suggest that circulating miR-21 may be a new biomarker of inflammation [54]. Interestingly, non monotonic age-related changes of miR-21 was observed in different age-groups of healthy subjects. Mir-21 levels increased mainly in octogenarian than in centenarins compared to younger subjects, according with the presence of an highest chronic inflammation levels in this setting of subjects [54]. Circulating miR-21 levels were correlated with C-reactive protein and fibrinogen levels, two well established biomarkers of inflammation [54].

### MiR-126

Tissue inflammation is critically regulated by miR-126 both in vitro and in vivo, via modulation of the expression of cell adhesion proteins, i.e. VCAM-1 [55]. Mice with knockdown of miR-126 function showed a renal inflammation, suggesting that miR-126 plays a major role in the response of renal microvascular endothelial cells to systemic inflammatory stimuli [55]. Accordingly, miR-126 is also deregulated in several disorders characterized by endothelial cell activation in response to systemic inflammatory stimuli, including cardiovascular diseases, diabetes mellitus and others inflammatory diseases [56]. Interestingly, miR-126 can down-regulates the expression of IKBβ, an important inhibitor of NF-κB signaling pathway [56].

### Other miRs

Other miRs were reported to be direct or indirect regulators of molecules belonging to TLRs pathway. MiR-147, was induced upon stimulation of multiple TLRs and functioned as a negative regulator of TLR-associated signaling events in murine macrophages [57]. Recently, TLR-4 activation, has been shown to down-regulate miR-107 expression in macrophages. In addition, miR-107 has been demonstrated to be deregulated in murine and rodent models of obesity and insulin resistance, contributing to both conditions [58]. Under cell cycle arrest conditions, miR-511 was reported to function as a positive regulator of TLR-4, whereas under replicative condition miR-511 seems to inhibit TLR-4 expression in monocytes and dendritic cells [59]. Recently, miR-200b and miR-200c were identified as factors that modify the efficiency of TLR-4 signaling through the MyD88-dependent pathway and can thus affect host innate defences against microbial pathogens [60]. Recent data also demonstrated that miR-187 directly targets TNF-α mRNA and indirectly decreases IL-6 and IL-12p40 expression via down-modulation of IκBζ, a master regulator of the transcription of these latter two cytokines. These results uncover a miRNA-mediated pathway controlling cytokine expression and demonstrate a central role of miR-187 in the physiological regulation of IL-10-driven anti-inflammatory responses [61].

Unconventional role of miRNAs were recently identified. Since miRNAs are short single strand RNA molecules, they can mimic viral RNA and consequently they can bind directly to TLRs 7–9, able to detect pathogenic nucleic acids. MiR let-7 is an highly abundant regulator of gene expression in the central nervous system and it is not only expressed in microglia, the major immune cells of the brain, but also in neurons. It was recently showed that extracellular let-7 activates the RNA-sensing TLR-7 and induces neurodegeneration in patients with Alzheimer’s disease [62]. A similar mechanism of miRNA-induced TLR activation was reported for miR-21: miR-21 can directly bind to TLR-7 in mice and TLR-8 in human macrophages, inducing the secretion of TNF-α and IL-6 [63]. Thus, miRNAs can function as agonists of the single-stranded RNA-binding TLRs, leading to NF-κB signaling activation and secretion of pro-inflammatory cytokines [64]. It can be hypothesized that these mechanisms could be much more widespread than what has been so far observed.

Overall, most of the miRNAs targeting TLRs pathway molecules act thought a negative feedback loop aimed to restrain the excessive pro-inflammatory response induced by TLR signaling activation. Consequently, a deregulation of miRNAs targeting TLR signaling molecules, may contribute to an inflammatory-antiinflammatory imbalance. Moreover, miRs can induce a chronic TLRs pathway activation binding directly to RNA-sensible TLRs. As a consequences, miR-induced TLRs deregulation could contribute to the development and progression of many inflammatory diseases, such as the major age-related diseases, including cardiovascular diseases, diabetes, Alzheimer and cancers [65].

### Cellular senescence and inflammaging: which role for TLRs pathway activation?

**Figure 2:**
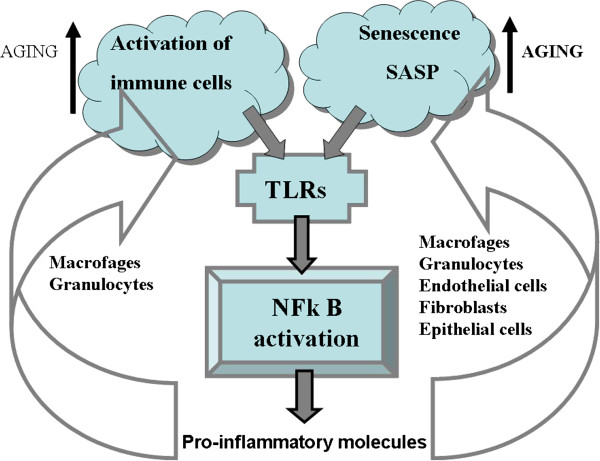
**NF**-**kB activation via TLRs can be induced by the activation of immune cells and by the acquisition of senescent phenotypes (SASP).**

Aging, and especially human aging, can be explained by the emerging concept of a combination of inflammation and aging. Aging, either physiological or pathological, can be driven by pro-inflammatory cytokines and substances produced by the innate immune system. The source of this chronic inflammation was mainly attributed to the progressive activation of immune cells over time [23]. However, recent studies have shown that the process of cellular senescence might be itself an important additional contributor to chronic inflammation [36,66,67]. Senescent cells can acquire a specific phenotype named “senescence-associated secretory phenotype” (SASP), characterized by the enhanced secretion of modulators of inflammatory status [66]. Interestingly, this phenomenon was documented not only in “immune cells” like macrophages [67,68], but also in fibroblast [66] and endothelial cells [36,69] (Figure 2).

Emerging data revealed that NF-κB signaling is the major pathway which stimulates the appearance of SASP [70,71]. There is a large number of NF-κB activators, and among them TLR ligands are the most potent [72,73]. In addition, TNF-α, IL-1, the antigen receptors found on the adaptive immune cells, specifically the T-cell receptor and B-cell receptor (TCR and BCR), and receptors found on antigen presenting cells CD40R can activate TLRs signaling. Furthermore, NF-κB can be activated by growth factors such as hepatocyte growth factor (HGF), follicle stimulating hormone (FSH), granulocyte macrophage-colony stimulating factor (GM-CSF), and nerve growth factor (NGF) and following DNA damage via mutation of the DNA response protein ataxia telangiectasia mutated (ATM), involving NEMO, that activates cytoplasmic IKK [74]. Genomic instability evoked by cellular stress triggers epigenetic changes, e.g. release of HMGB1 proteins which are also potent enhancers of inflammatory responses. Moreover, environmental stress and chronic inflammation can stimulate p38MAPK and ceramide signaling and induce cellular senescence with pro-inflammatory responses [74]. On the other hand, two cyclin-dependent kinase inhibitors, p16INK4a and p14ARF, are effective inhibitors of NF-κB signaling.

Even if many data confirmed the relationship between SASP and NF-κB signaling activation, few data were reported on the molecular relationship between TLRs pathway activation and SASP. We recently reported that the most hyper-expressed miRNAs in senescent HUVEC compared to younger cells, target TLRs signaling pathway, suggesting that a deregulation of TLRs pathway is directly or indirectly associated with cellular senescence and acquisition of SASP [36]. At the cellular level, both proliferative and/or oxidative-stress induced cell senescence associated with a pro-inflammatory state may greatly contribute to inflammaging [75]. It was demonstrated that TLR-2 translates oxidative tissue damage into inflammatory responses by mediating the effects of oxidized phospholipids [76]. Thus, it can be hypothesized that chronic activation of TLRs by endogenous ligands, i.e. oxidized phospholipids, in absence of pathogens infection, can promote the acquisition of SASP both in vivo and in in vitro cultured cells during replicative senescence.

Overall, we hypothesize that different mechanisms can contribute to perpetuation of inflammaging: i) the progressive activation of immune cells over time, ii) the ineffective clearance of pathogens by aged macrophages that can increase the duration of their activation via TLRs, iii) endogenous TLRs ligands released by senescent cells that can contribute to chronic TLR activation in absence of pathogens infection, iiii) miRNAs hyper-expressed and released by senescent cells that can directly activate TLRs in cells of innate immunity and in endothelial cells.

## Conclusion

The aging process is associated with a loss of complexity in the dynamics of physiological systems that reduce the ability to adapt to stress, causing frailty and/or age-related diseases.

An increase of inflammatory markers was described as a general feature of the ageing process, and thus named as inflammaging. Inflammaging phenotype is a complex phenotype which is the result of the age-related cell/tissue adaptation and remodelling, involving not only innate but also adaptive immunity, as well as other tissues or organs (gut, fat tissue, liver, muscle, brain). Importantly, inflammaging appears accelerated in many age-associated diseases. The source of the age-associated chronic inflammation was mainly attributed to the progressive activation of immune cells over time and to the acquisition of SASP. Thus, the accumulation of senescent cells in aged subjects could contribute to the perpetuation of inflammaging, and the systemic chronic inflammatory status could, in turn, contribute to the diseases development. Both phenomena, the activation of immune cells over time and the accumulation of senescent cells in aged subjects, are strictly related to TLRs activation. An altered TLRs/ligands interaction seems to contribute to the impairment of reparative and regenerative capability of the aged tissues, in normal aging and mainly in age-related diseases. Increasing evidence suggests that miRNAs could play important roles in this scenario, modulating both TLRs pathway activation and SASP acquisition. MiRNAs can have two opposite roles: TLR activation and NF-kB signalling inhibition, in a complex scenario where low and chronic inflammation prevails, likely also sustained by cell senescence secretome. MiRNAs inhibition effect probably belongs to the different levels of anti-inflammatory pathways that have evolved to abate TLR signalling to prevent cell and tissue destruction [77].

To improve our understanding of the contribution of cellular senescence/SASP to ageing and age-related disease, it is imperative to define a specific signature of cellular senescence that is functionally connected with normal and pathological ageing. Currently, there are many gaps in our knowledge of the processes leading to senescence, and the signature of cellular senescence both in vitro and in vivo [78]. In conclusion, the identification of a well-defined set of senescence biomarkers for ageing and age-related disease could have a strong impact on the diagnosis, staging and predicted outcomes of age-related disease, providing the basis for a pharmacological intervention to postpone ageing and age-related disease.

## Competing interest

None of the authors have any conflict of interest.

## Authors’ contributions

All of the authors contributed significantly to the work: conception and drafting of the manuscript: FO and MRR; (2) collection of literature data and critical interpretation: FP and LB; and (3) revising the manuscript for intellectual content: RR, LG and ADP. All authors read and approved the final manuscript.

## Research funding

The present study was supported by grants from the Università Politecnica delle Marche to ADP, MRR and FO.
